# Multivessel Intervention in Myocardial Infarction with Cardiogenic Shock: CULPRIT-SHOCK Trial Outcomes in the PL-ACS Registry

**DOI:** 10.3390/jcm10091832

**Published:** 2021-04-22

**Authors:** Mariusz Gąsior, Piotr Desperak, Dariusz Dudek, Adam Witkowski, Paweł E. Buszman, Przemysław Trzeciak, Michał Hawranek, Marek Gierlotka, Stanisław Bartuś, Marek Grygier, Michał Zembala, Janina Stępińska, Jacek Legutko, Wojciech Wojakowski

**Affiliations:** 1 Third Department of Cardiology, Faculty of Medical Sciences in Zabrze, Medical University of Silesia, 40-752 Katowice, Poland; desperak.piotr@gmail.com (P.D.); przemyslaw.t@wp.pl (P.T.); mhawranek@poczta.fm (M.H.); 2 Department of Cardiology, University Hospital, Faculty of Medicine, Institute of Cardiology, Jagiellonian University Medical College, 31-007 Krakow, Poland; mcdudek@cyfronet.pl (D.D.); sbartus@cathlab.krakow.pl (S.B.); 3 Department of Interventional Cardiology and Angiology, National Institute of Cardiology, 04-628 Warsaw, Poland; witkowski@hbz.pl; 4 Department of Epidemiology and Statistics, Medical School of Silesia, 40-752 Katowice, Poland; pbuszman@ka.onet.pl; 5 Department of Cardiology, University Hospital, Institute of Medical Sciences, University of Opole, 45-060 Opole, Poland; marek.gierlotka@gmail.com; 6 First Department of Cardiology, Poznan University of Medical Sciences, 61-701 Poznan, Poland; mgrygier@wp.pl; 7 Department of Cardiac Surgery, Heart and Lung Transplantation and Mechanical Circulatory Support, Silesian Center for Heart Diseases, 41-800 Zabrze, Poland; m.zembala.jr@sccs.pl; 8 Department of Intensive Cardiac Therapy, National Institute of Cardiology, 04-628 Warsaw, Poland; janina@stepinska.pl.pl; 9 Department of Interventional Cardiology, John Paul II Hospital, Faculty of Medicine, Institute of Cardiology, Jagiellonian University Medical College, 31-007 Krakow, Poland; jlegutko@kcri.org; 10 Department of Cardiology and Structural Heart Diseases, Medical University of Silesia, 40-752 Katowice, Poland; wojtek.wojakowski@gmail.com

**Keywords:** acute myocardial infarction, cardiogenic shock, percutaneous coronary intervention

## Abstract

Background: The aim of the study was a comparison of culprit-lesion-only (CL-PCI) with the multivessel percutaneous coronary intervention (MV-PCI) in terms of 30-day and 12-month mortality in a national registry. Methods: Patients from the PL-ACS registry with MI and CS were analyzed. Patients meeting the criteria of the CULPRIT-SHOCK trial were divided into two groups: CL-PCI and MV-PCI groups. Results: Of the 3265 patients in the PL-ACS registry with MI complicated by CS, the criteria of the CULPRIT-SHOCK trial were met by 2084 patients (63.8%). The CL-PCI was performed in 883 patients, and MV-PCI was performed in 1045 patients. After the propensity score matching analysis, 617 well-matched pairs were obtained. In a 30-day follow-up, death from any cause occurred in 49.3% in the CL-PCI group and 57.0% in the MV-PCI group (RR 0.86, 95% CI 0.58–0.92, *p* = 0.0081). After 12 months, the rate of mortality was 62.5% in the CL-PCI group and 68.0% in the MV-PCI group (RR 0.92, 95% CI 0.84–1.01, *p* = 0.066). Conclusions: The results confirm the validity of CULPRIT-SHOCK findings in a national registry and current guideline-recommended strategy of revascularization limited to the infarct-related artery.

## 1. Introduction

Left ventricular failure and cardiogenic shock (CS) developing in its course determine the prognosis among patients with acute myocardial infarction (AMI) [1,2]. The only effective treatment for CS is emergency revascularization aimed to restore normal myocardial perfusion to the area at risk of necrosis [3,4,5]. The majority of patients with CS have multivessel coronary artery disease (CAD) [5]. Both the presence of multivessel CAD and an angiographic location of the infarction-related artery are associated with adverse prognosis [6,7,8]. It has been known that cardiac output in patients with CS is also maintained by compensatory hyperkinesia of non-necrotic segments. If hemodynamically significant stenosis is found in non-infarct-related coronary arteries, limited perfusion may reduce or even eliminate this effect, leading to further deterioration of left ventricular function.

Therefore, the single-stage multivessel revascularization of all significant lesions had its strong supporters despite the known limitations (e.g., additional traumatization of coronary arteries, acute ischemia of area not-at-risk of cardiac necrosis, higher contrast volume with depressive effect on kidney function). Previous observational studies gave conflicting results of an immediate, multivessel percutaneous coronary intervention (MV-PCI) [7,9,10,11,12,13,14,15,16,17]. The systematic review and meta-analysis of de Waha et al. did not confirm the benefits of immediate MV-PCI, which was associated with higher short-term mortality in comparison to the PCI of the culprit-lesion-only (CL-PCI) [18]. Published results of the first international, multicenter, randomized trial PCI Strategies in Patients with Acute Myocardial Infarction and Cardiogenic Shock (CULPRIT-SHOCK; ClinicalTrials.gov number: NCT01927549) showed a significant reduction in 30-day risk of a composite of death or severe renal failure, leading to renal-replacement therapy for CL-PCI compared to immediate MV-PCI [19]. The CULPRIT-SHOCK trial has radically changed the approach of the treatment strategy of AMI complicated by CS, resulting in a modification in the guidelines of the European Society of Cardiology [20,21]. However, a potential problem with some randomized clinical trials may be the difficulty in translating the evidence obtained into clinical practice.

Some randomized clinical trials often include highly selected patients who may not fully reflect the real-world population [22,23,24], which can also affect the benefits and risks of using novel drugs or technologies [25,26]. Current European Society of Cardiology guidelines recommended the necessity of continuous observation of patients to relate randomized clinical trials’ results to real-world populations [25,26]. Therefore, based on previous experience [27], we decided to compare CL-PCI with MV-PCI strategies in terms of 30-day and 12-month risk of death from any cause in the population of a large national registry.

## 2. Materials and Methods

### 2.1. The PL-ACS Registry and Data Sources

Data from the registry of the Polish Registry of Acute Coronary Syndromes (PL-ACS) were retrospectively analyzed [28]. The PL-ACS is a national, multicenter, ongoing, prospective observational registry, which includes data on patients hospitalized with ACS in Poland. In brief, the PL-ACS is a joint project of the Silesian Center of Heart Diseases in Zabrze and the Polish Ministry of Health, in cooperation with the National Health Fund. The registry was founded in October 2003, and in May 2004, the registry protocol was harmonized with the European Cardiology Audit and Registration Data Standards. Due to the obligatory angiographic data included in the study protocol, the analysis was undertaken in consecutive patients included in the registry in the calendar years 2008–2019. Data were collected by the treating physicians and entered into the electronic system of the registry. Data on follow-up mortality, including the date of death, were obtained from the National Health Fund.

In addition, the study uses data on after-discharge follow-up from the Polish Nationwide Acute Myocardial Infarction Database—AMI-PL [29], and the Silesian Cardiovascular Database—SILCARD [30]. The AMI-PL includes all cases of AMI that occurred between 2009 and 2014. The database contains the record of all AMI cases provided by the National Health Fund, the sole public and compulsory health insurer in Poland. The AMI cases are selected based on a primary diagnosis coded in the International Classification of Diseases, Tenth Revision, as I21 or I22, irrespective of any AMI occurrence in the past. The SILCARD database was based on the agreement between the Silesian Center for Heart Diseases in Zabrze and the Regional Department of National Health Fund in Katowice to conduct a comprehensive analysis of patients with cardiovascular diseases in the Silesian Province. General information on the SILCARD database was previously reported. Briefly, the database contains records from all hospitals (*n* = 310) in the Silesian Province—a large administrative region in Southern Poland with a population of 4.57 million (roughly 12% of Poland’s total population), of which 3.80 million are adults. The National Health Fund provided all data for the database, covering the period between 2006 and 2016. Cardiovascular disease was defined as R52 or J96 or any I code according to the 10th revision of the International Classification of Diseases.

The institutional review board at each site approved all protocols. The registries meet the conditions of the Declaration of Helsinki and its later amendments.

### 2.2. The Design and Results of the CULPRIT-SHOCK Trial

The design [31] and outcomes [19,32] of the CULPRIT-SHOCK trial have been described previously. In brief, the CULPRIT-SHOCK trial was an investigator-initiated, randomized, open-label, European multicenter trial of 706 patients with multivessel disease, AMI, and CS. The study population was randomly assigned to one of two initial revascularization strategies: the CL-PCI group or the MV-PCI group. The primary endpoint was a composite of death or severe renal failure leading to renal-replacement therapy within 30 days after randomization.

At 30 days, the composite primary endpoint of death or renal-replacement therapy had occurred in 45.9% in the CL-PCI group and in 55.4% in the MV-PCI group (relative risk (RR): 0.83, 95% confidence interval (CI): 0.71–0.96, *p* = 0.01) [19]. At 1 year, death had occurred in 50.0% in the CL-PCI group and in 56.9% in the MV-PCI group (RR: 0.88, 95% CI: 0.76–1.01) [32].

### 2.3. Study Population

For the purposes of the present study of all patients in the PL-ACS registry hospitalized between 2008 and 2019, only patients with AMI, complicated by CS on admission, with complete angiographic data, and treated with the percutaneous coronary intervention were included.

In the first step, we applied the CULPRIT-SHOCK inclusion to the study population:

1. Multivessel CAD defined as at least two major vessels (≥2 mm in diameter) with >70% stenosis of the diameter,
2. An identifiable culprit lesion.

Then, among the remaining patients from the study population from the PL-ACS registry, the CULPRIT-SHOCK exclusion criteria were adopted:

1. No intrinsic heart action,
2. Need for primary urgent coronary artery bypass grafting,
3. Mechanical cause of CS,
4. Massive pulmonary embolism,
5. Age >90 years,
6. Shock of other cause (bradycardia, sepsis, hypovolemia, etc.),
7. Other known severe concomitant disease with limited life expectancy <6 months,
8. Pregnancy,
9. Known severe renal insufficiency (creatinine clearance <30 mL/kg).

Data on the other exclusion criteria of the CULPRIT-SHOCK trial (onset of shock > 12 h, resuscitation > 30 min, cerebral deficit with fixed dilated pupils) were not verifiable in the PL-ACS registry. Therefore, they were not used in the analysis. 

Patients from the PL-ACS population who met the inclusion criteria and did not meet the exclusion criteria of the CULPRIT-SHOCK trial were the target study group, namely, the CULPRIT-SHOCK-like cohort. Remaining patients (did not meet the inclusion or met at least one of the exclusion criteria of the CULRPIT-SHOCK trial) were assigned to the CULPRIT-SHOCK-not-fulfilling cohort. 

In the next step, the CULPRIT-SHOCK-like cohort was divided into two groups depending on the revascularization strategy: either PCI of the culprit-lesion only with the option of staged revascularization of non-culprit lesions (the CL-PCI group), or immediate multivessel PCI (the MV-PCI group). 

### 2.4. Diagnostics and Treatment

The management of the study population was in accordance with contemporary recommendations of the European Society of Cardiology in AMI [20,21]. Briefly, during or after transfer to the hospital, the loading doses of acetylsalicylic acid, P2Y12 inhibitor, and weight-adjusted unfractionated heparin were administrated. In all cases, coronary angiography with standard techniques and equipment was performed. The decision on the access site (radial, femoral, or other) and the type of diagnostic catheter was made by the operator. All therapeutic decisions after coronary angiography, balloon pre-dilatation and post-dilatation, use of stents, type of stents, glycoprotein IIb/IIIa receptor inhibitors, use of mechanical circulatory support, and other established interventional techniques were at the operator’s discretion. After the procedure, the patients were transferred to the intensive care unit. In case of recurrence of ischemia, urgent coronary angiography was performed. After discharge, dual antiplatelet therapy was recommended for at least 12 months. Furthermore, each patient has been prescribed standard secondary prevention in accordance with the contemporary guidelines.

### 2.5. Definitions and Outcome Measures

CS in PL-ACS was defined with a generally accepted definition as a systolic blood pressure <90 mmHg or the use of catecholamine therapy to maintain a systolic pressure of at least 90 mmHg, clinical signs of pulmonary congestion, and signs of impaired organ perfusion with at least one of the following manifestations: altered mental status, cold and clammy skin and limbs, oliguria with a urine output <30 mL per hour, or an arterial lactate level > 2.0 mmol per liter. Multivessel CAD was defined as a significant stenosis in left main (LM) or in at least two major epicardial territories or in their major branches (left anterior descending (LAD), left circumflex, or right coronary artery system). A significant lesion was defined as ≥50% diameter stenosis or positive result of fractional flow reserve in vessels with a diameter ≥2.0 mm. Due to the lack of data on the exact hour of the procedure in the PL-ACS registry, the MV-PCI group was defined as an intervention in at least two major vessels on the same day. Study design and analysis were based on per-protocol analysis.

The primary outcome measure included the occurrence of death from any cause within 30 days and at 12 months after admission. 

### 2.6. Statistical Analysis

Analyzed variables are expressed as numbers and percentages. Analogous to the CULRPIT-SHOCK trial [19], continuous variables were summarized using arithmetic a median with quartiles 1 and 3. The normality of the distribution was verified using the Shapiro–Wilk test. The analysis of Student’s *t*-test for comparison of continuous parameters with normal distribution was performed, whereas the Mann–Whitney U test for parameters with non-normal distribution was used. Categorical variables were compared using the Chi-square Pearson’s test (with the Yates correction if the expected number of observations was less than 5). All-cause mortality in 12-month follow-up was analyzed using the Kaplan–Meier method with the log-rank test. 

To minimize the confounding impact of risk factors affecting death from any cause in 30 days and 12 months, we performed the propensity score analysis to adjust for differences in patients’ baseline characteristics. First, the logistic regression was performed to score all patients according to the treatment (the CL-PCI group versus the MV-PCI group) using as covariates clinical and procedural parameters that were clinically relevant for the endpoint: age, arterial hypertension, diabetes mellitus, diastolic blood pressure on admission, heart rate on admission, hypercholesterolemia, initial Thrombolysis in Myocardial Infarction (TIMI) grade flow of culprit lesion, PCI of left circumflex, PCI LAD, PCI of LM, PCI of right coronary artery, previous coronary artery bypass grafting, previous PCI, resuscitation before PCI, sex, ST-segment elevation AMI on admission, and systolic blood pressure on admission. In the next stage, the analyses were performed on the study groups, stratified by pairs to account for propensity score matching. The forced matching algorithm was used for variables that most differed in the initial characteristics: arterial hypertension, PCI LM, PCI LAD, PCI of right coronary artery, resuscitation before PCI, and initial TIMI grade flow of culprit lesion. The Greedy nearest neighbor matching without replacement was used. A two-sided *p*-value <0.05 was considered significant. The NCSS 2019 Statistical Software (NCSS, LLC., Kaysville, UT, USA, ncss.com/software/ncss, accessed on 20 May 2020) was used for all calculations.

## 3. Results

### 3.1. Identification of the CULPRIT-SHOCK-Like Cohort

**Figure 1:**
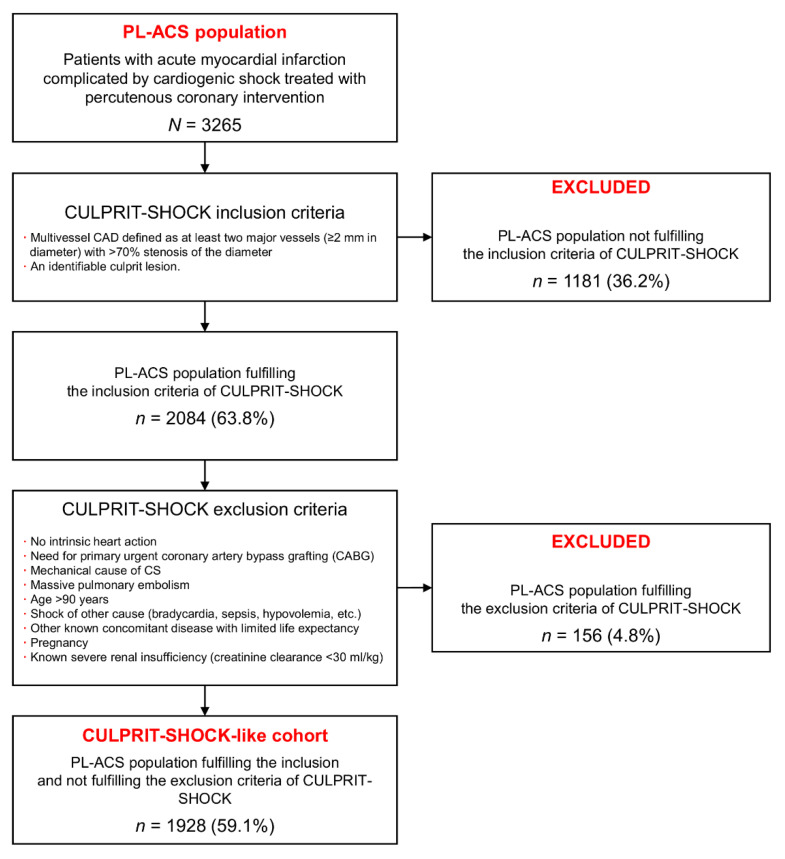
Flow chart for identification of the CULPRIT-SHOCK-like group in the PL-ACS registry population based on the CULPRIT-SHOCK trial inclusion and exclusion criteria.

A flow chart for the identification of the CULPRIT-SHOCK criteria in the CULPRIT-SHOCK-like cohort of the PL-ACS population is presented in Figure 1. Among 3265 patients with AMI, CS, and treated with PCI from PL-ACS, the inclusion criteria of the CULPRIT-SHOCK trial were met in 2084 (63.8%). 

Subsequently, among the 2084 patients from the study population who met the inclusion criteria, the CULPRIT-SHOCK trial exclusion criteria were applied. Therefore, 156 patients (4.8%) were further excluded. In summary, in the study population, the CULPRIT-SHOCK criteria have been met in 1928 patients (59.1%) (CULPRIT-SHOCK-like cohort), and the remaining 1337 (40.9%) patients did not meet the criteria (the CULPRIT-SHOCK-not-fulfilling cohort).

The reasons for non-inclusion in our study were: single-vessel CAD (*n* = 1033 patients; 31.6%) and the inability to identify a culprit lesion (*n* = 148 patients; 4.5%). On the other hand, the most common exclusion criteria in the study population were: age > 90 years (*n* = 45; 1.4%), known severe renal insufficiency (*n* = 34; 1.0%), mechanical cause of CS (*n* = 32; 1.0%), need for urgent coronary artery bypass grafting (*n* = 26; 0.8%), and no intrinsic heart action (*n* = 25; 0.8%).

### 3.2. Comparison of the CL-PCI versus the MV PCI

In the CULPRIT-SHOCK-like cohort, baseline characteristics and treatment outcomes of patients undergoing CL-PCI versus MV-PCI were compared. The mean age of the CULPRIT-SHOCK-like cohort was 69.5 ± 10.9 years, and 37.1% were female. The CL-PCI was performed in 883 (45.8%) patients, whereas the MV-PCI was performed in the remaining 1045 (54.2%) patients. Baseline characteristics of the study groups are summarized in Table 1 and Table 2. Current smokers, arterial hypertension, hypercholesterolemia, and prior coronary artery bypass grafting were more prevalent in patients treated with the CL-PCI in comparison to the MV-PCI. They were also more often resuscitated before PCI. In angiographic characteristics, the CL-PCI group had less frequent PCI of LM, more often PCI of right coronary artery, and bypass graft, and poorer TIMI grade flow in comparison to the MV-PCI group. Regarding therapy, stent implantation, mechanical circulatory support, and glycoprotein IIb/IIIa inhibitors were used less frequently in the CL-PCI than in the MV-PCI group.

Table 3 contains the 30-day and 12-month outcomes. In a 30-day follow-up, death of any cause occurred in 49.1% in the CL-PCI group and 54.8% in the MV-PCI group (RR: 0.90, 95% CI: 0.82–0.98, *p* = 0.015). In the remaining, limited data of 30-day outcomes, recurrent myocardial infarction and staged or urgent repeat revascularization were more frequent in the CL-PCI. After 12 months, the incidence of death of any cause was 61.7% in the CL-PCI group and 66.1% in the MV-PCI group (HR 0.93, 95% CI 0.86–1.01, *p* = 0.075). The incidence of recurrent myocardial infarction, rehospitalization for congestive heart failure, and stroke was similar in both groups. Either percutaneous or surgical repeat revascularization was higher in patients treated with the CL-PCI than the MV-PCI.

### 3.3. Comparison of the CL-PCI versus the MV PCI in the Matched Cohort

**Figure 2:**
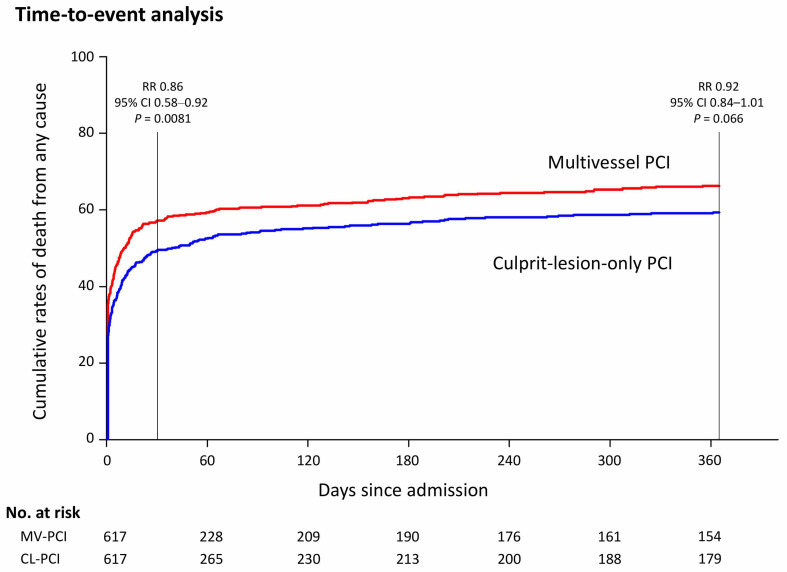
Event rates of the death from any cause at 12 months in study groups. Abbreviations: CI = confidence interval; CL = culprit-lesion-only; HR = hazard ratio; MV = multivessel; PCI = percutaneous coronary intervention.

After the propensity score matching of study groups, 617 pairs of patients were selected with no differences in baseline and angiographic characteristics (Table 1 and Table 2). Kaplan–Meier curves in analyzed matched groups are presented in Figure 2. In a 30-day follow-up, the incidence of death of any cause was 49.3% in the CL-PCI group and 57.0% in the MV-PCI group (RR: 0.86, 95% CI: 0.58–0.92, *p* = 0.0081). At 12 months, all-cause mortality was 61.7% in the CL-PCI group and 66.1% in the MV-PCI group (RR 0.92, 95% CI 0.84–1.01, *p* = 0.066). Besides more frequent revascularization in the CL-PCI group, no other significant differences between both groups in the 30-day and 12-month follow-up were found.

## 4. Discussion

### 4.1. CULPRIT-SHOCK Criteria

The analysis of the prospective, national PL-ACS registry including real-world population showed that over 59% of patients with MI complicated by CS met the CULPRIT-SHOCK criteria. The remaining patients were not analyzed due to non-compliance with the inclusion criteria (over 36%) or met the exclusion criteria (less than 5%) of the CULPRIT-SHOCK study. Despite the higher level of evidence than non-randomized studies, randomized clinical trials may not always reflect the real-world patients. This may affect the benefits and risks of using the novel therapeutic strategies in daily clinical practice [22,23,24,25,26]. The percentage of PL-ACS patients meeting the criteria is consistent with the number of patients screened for participation in the CULPRIT-SHOCK trial (66%; 1075 in screening, 706 enrolled in randomization) [32]. 

### 4.2. Strategies of Revascularization

Of 1928 included patients with AMI complicated by CS and multivessel CAD, 883 (46%) underwent CL-PCI, while the remaining 1045 (54%) underwent MV-PCI. In the largest contemporary trials and registers, the reported percentage of patients undergoing MV-PCI was 14% to 37% (numerically 60 to 433 patients) [9,14,15,16,17]. Therefore, our study concerns a large number of patients with a very high percentage of MV-PCI. This result deserves careful discussion because it is a non-randomized analysis of the applied treatment strategy (per-protocol). The decision on MV-PCI may be conditioned by various factors, e.g., coronary arteries’ anatomy, the complexity of PCI, predicted final effect of the intervention, hemodynamic status, type of myocardial infarction, other organ function, or other clinical and angiographic factors.

Detailed analysis showed that patients undergoing MV-PCI in comparison to the CL-PCI group have a less frequent presence of coronary artery disease risk factors, a history of cardiac surgical revascularization, an incidence of pre-hospital cardiac arrest, and more often a higher heart rate on admission, a PCI of LM, stent implantation, a final TIMI grade 3 flow in infarct-related artery, and use of glycoprotein IIb/IIIa inhibitors. It is difficult to determine which factors had a decisive impact on the revascularization strategy because, depending on their frequency, they may balance each other out in both groups. Nevertheless, qualitatively, predictors of adverse prognosis were more common in the CL-PCI group, which, compared to other non-randomized studies, could have a significant impact on the choice of treatment strategy and outcomes [18]. Therefore, in the initial study population, we can only correctly determine the factors that had an impact on operators’ decisions, without specifying specific conclusions about which treatment strategy improves prognosis.

### 4.3. Comparison of Treatment Outcomes Depending on Revascularization Strategy

Therefore, the next step in our study was to perform a propensity score matching analysis, in which 617 pairs of patients were selected. Propensity score matching allowed reducing the differences in baseline characteristics between the study groups. The CL-PCI compared to the MV-PCI strategy was associated with a lower risk of 30-day death from any cause, whereas at 12 months, no significant difference between both groups was found. Presented results obtained from large, nationwide registry confirmed the outcomes of the CULPRIT-SHOCK trial [19,32]. Additionally, they are consistent with the meta-analysis of de Waha et al. including 10 studies (6051 patients), which showed higher short-term mortality in the MV-PCI group, without a significant difference in the 12-month follow-up [18].

It is noteworthy that in our study, the overall percentage of death from any cause was higher in comparison to the CULPRIT-SHOCK trial. The reason for the above fact may be significant differences in baseline characteristics between the PL-ACS and the CULPRIT-SHOCK populations. In PL-ACS patients, a higher incidence of ST-elevation AMI and initial TIMI grade 0/1 flow, a lower final TIMI grade 3 flow, and use of mechanical circulatory support were observed.

### 4.4. Study Limitation

We did not have information on the initial revascularization strategy planned by the operator (intention-to-treat). Data on severe renal failure leading to renal-replacement therapy within 30-day follow-up was not available. The present study inclusion period (2008–2019) was different from the recruitment period for the CULPRIT-SHOCK trial (2013–2016), which could have had a significant impact on the treatment strategy. Therefore, the results of our study should be interpreted with caution.

## 5. Conclusions

We confirmed the validity of CULPRIT-SHOCK findings in a large, all-comers registry. The results of our study confirm the current guideline-recommended strategy of treatment limited to the infarct-related artery in the real-world population.

## Figures and Tables

**Table 1:** Baseline characteristics of study groups of the PL-ACS registry and the CULPRIT-SHOCK trial.

Parameter	PL-ACS Population *N* = 1928			PL-ACS Matched Cohort *N* = 1234		
	Culprit-Lesion-Only PCI *n* = 883	Multivessel PCI *n* = 1045	*p*	Culprit-Lesion-Only PCI *n* = 617	Multivessel PCI *n* = 617	*p*
Age, years, median (Q1–Q3)	70 (62–78)	69 (61–78)	0.17	70 (62–78)	69 (62–78)	0.55
Male sex, *n*/*N* (%)	535/883 (60.6)	677/1045 (64.8)	0.057	380/617 (61.6)	397/617 (64.3)	0.32
Weight, kg, median (Q1–Q3)	80 (70–88)	78 (70–88)	0.37	80 (70–87)	79 (70–87)	0.37
Height, cm, median (Q1–Q3)	170 (164–175)	170 (164–175)	0.37	170 (164–175)	170 (164–175)	0.42
BMI, kg/m^2^, median (Q1–Q3)	27 (25–30)	27 (24–29)	0.069	27 (25–30)	27 (25–30)	0.063
Cardiovascular risk factors						
Current smoking, *n*/*N* (%)	220/691 (31.8)	256/946 (27.1)	0.036	169/513 (32.9)	153/543 (28.2)	0.093
Arterial hypertension, *n*/*N* (%)	506/789 (64.1)	567/997 (56.9)	0.0019	358/580 (61.7)	358/580 (61.7)	>0.99
Hypercholesterolemia, *n*/*N* (%)	289/753 (38.4)	330/984 (33.5)	0.037	198/565 (35.0)	192/574 (33.4)	0.57
Diabetes mellitus, *n*/*N* (%)	284/800 (35.5)	308/989 (31.1)	0.051	192/584 (32.9)	186/584 (31.8)	0.71
Previous MI, *n*/*N* (%)	185/805 (23.0)	212/1007 (21.1)	0.32	120/586 (20.5)	117/593 (19.7)	0.75
Previous PCI, *n*/*N* (%)	105/810 (13.0)	129/1009 (12.8)	0.91	63/588 (10.7)	69/595 (11.6)	0.63
Previous CABG, *n*/*N* (%)	48/818 (5.9)	27/1007 (2.7)	0.0006	20/591 (3.4)	13/593 (2.2)	0.21
Fibrinolysis before PCI	1/544 (0.2)	6/829 (0.7)	0.32	1/452 (0.2)	3/445 (0.7)	0.60
Resuscitation before PCI, *n*/*N* (%)	469/882 (53.2)	416/1044 (39.8)	<0.0001	322/617 (52.2)	322/617 (52.2)	>0.99
STEMI, *n*/*N* (%)	637/883 (72.1)	715/1045 (68.4)	0.075	441/617 (71.5)	429/617 (69.5)	0.45
LBBB, *n*/*N* (%)	59/881 (6.7)	91/1044 (8.7)	0.10	47/615 (7.6)	57/617 (9.2)	0.31
SBP, mmHg, median (Q1–Q3)	89 (70–100)	89 (70–105)	0.84	90 (70–100)	88 (70–100)	0.92
DBP, mmHg, median (Q1–Q3)	60 (40–67)	60 (40–70)	0.61	60 (40–65)	60 (40–70)	0.66
Heart rate, bpm, median (Q1–Q3)	80 (65–100)	88 (70–102)	0.016	83 (70–105)	85 (70–100)	0.74
Creatinine, mg/dL, median (Q1–Q3)	1.20 (0.94–1.52)	1.25 (1.01–1.49)	0.56	1.17 (0.92–151)	1.29 (1.04–1.60)	0.14
No. of affected vessels						
1, *n*/*N* (%)	-	-		-	-	
2, *n*/*N* (%)	436/883 (49.4)	505/1045 (48.3)	0.65	299/617 (48.5)	320/617 (51.9)	0.23
3, *n*/*N* (%)	447/883 (50.6)	540/1045 (51.7)	0.65	320/617 (51.9)	299/617 (48.5)	0.23
Vessel related to the infarction						
LM, *n*/*N* (%)	67/883 (7.6)	228/1045 (21.8)	<0.0001	65/617 (10.5)	65/617 (10.5)	>0.99
LAD, *n*/*N* (%)	353/883 (40.0)	463/1045 (44.3)	0.055	289/617 (46.8)	289/617 (46.8)	>0.99
Cx, *n*/*N* (%)	142/883 (16.1)	149/1045 (14.3)	0.27	91/617 (14.7)	96/617 (15.6)	0.69
RCA, *n*/*N* (%)	305/883 (34.5)	203/1045 (19.4)	<0.0001	165/617 (26.7)	165/617 (26.7)	>0.99
Bypass graft, *n*/*N* (%)	16/883 (1.8)	2/1045 (0.2)	0.0014	7/617 (1.1)	2/617 (0.3)	0.18
LVEF, *n*/*N* (%)	35 (25–45)	34 (25–41)	0.051	35 (25–45)	35 (25–43)	0.45

Abbreviations: BMI = body mass index; CABG = coronary artery bypass grafting; Cx = circumflex artery; DBP = diastolic blood pressure; LAD = left anterior descending artery; LBBB = left bundle-branch block; LM = left main; LVEF = left ventricular ejection fraction; MBP = mean blood pressure; MI = myocardial infarction; PCI = percutaneous coronary intervention; Q1–Q3 = quartiles 1 and 3; RCA = right coronary artery; SBP = systolic blood pressure; STEMI = ST-segment elevation myocardial infarction.

**Table 2:** Procedural characteristics of study groups of the PL-ACS registry and the CULPRIT-SHOCK trial.

Parameter	PL-ACS Population *N* = 1928			PL-ACS Matched Cohort *N* = 1234		
	Culprit-Lesion-Only PCI *n* = 883	Multivessel PCI *n* = 1045	*p*	Culprit-Lesion-Only PCI *n* = 617	Multivessel PCI *n* = 617	*p*
Stent in culprit lesion						
Any, *n*/*N* (%)	750/883 (84.9)	929/1045 (88.9)	0.0098	541/617 (87.7)	545/617 (88.3)	0.75
Drug eluting, *n*/*N* (%)	454/750 (60.5)	529/929 (56.9)	0.14	296/541 (54.7)	305/545 (56.0)	0.68
TIMI grade for blood flow						
Before PCI of culprit lesion						
0, *n*/*N* (%)	353/545 (64.8)	446/844 (52.8)		277/452 (61.3)	277/452 (61.3)	
I, *n*/*N* (%)	97/545 (17.8)	169/844 (20.0)		89/452 (19.7)	89/452 (19.7)	
II, *n*/*N* (%)	49/545 (9)	126/844 (14.9)		45/452 (10.0)	45/452 (10.0)	
III, *n*/*N* (%)	46/545 (8.4)	103/844 (12.2)	<0.0001	41/452 (9.1)	41/452 (9.1)	>0.99
After PCI of culprit lesion						
0, *n*/*N* (%)	66/856 (7.7)	61/1025 (6.0)		43/602 (7.1)	35/603 (5.8)	
I, *n*/N (%)	45/856 (5.3)	45/1025 (4.4)		37/602 (6.1)	33/603 (5.5)	
II, *n*/*N* (%)	94/856 (11.0)	77/1025 (7.5)		67/602 (11.1)	44/603 (7.3)	
III, *n*/*N* (%)	651/856 (76.1)	842/1025 (82.1)	0.011	455/602 (75.6)	491/603 (81.4)	0.066
Staged PCI of nonculprit lesions, *n*/*N* (%)	120/883 (13.6)	44/1045 (4.2)	<0.0001	89/617 (14.4)	31/617 (5.0)	<0.0001
Mechanical circulatory support, *n*/*N* (%)	131/879 (14.9)	204/1045 (19.5)	0.0078	105/615 (17.1)	119/617 (19.3)	0.31
GP IIb/IIIa inhibitor, *n*/*N* (%)	343/874 (39.2)	468/1042 (44.9)	0.012	242/613 (39.5)	275/615 (44.7)	0.063

Abbreviations: GP = glycoprotein; PCI = percutaneous coronary intervention; TIMI = Thrombolysis in Myocardial Infarction.

**Table 3:** Procedural characteristics of study groups of the PL-ACS registry and the CULPRIT-SHOCK trial.

Parameter	PL-ACS Population *N* = 1928				PL-ACS Matched Cohort *N* = 1234			
	Culprit-Lesion-Only PCI *n* = 883	Multivessel PCI *n* = 1045	Relative Risk (95% CI)	*p*	Culprit-Lesion-Only PCI *n* = 617	Multivessel PCI *n* = 617	Relative Risk (95% CI)	*p*
Outcomes at 30 days								
Death from any cause, *n*/*N* (%)	396/807 (49.1)	554/1011 (54.8)	0.90 (0.82–0.98)	0.015	289/586 (49.3)	337/591 (57.0)	0.86 (0.58–0.92)	0.0081
Recurrent myocardial infarction, *n*/*N* (%)	9/248 (3.6)	7/663 (1.1)	3.44 (1.19–10.14)	0.0085	6/192 (3.1)	3/356 (0.8)	3.80 (0.84–19.6)	0.098
Rehospitalization for congestive heart failure, *n*/*N* (%)	3/248 (1.2)	6/663 (0.9)	1.34 (0.27–5.94)	0.68	3/192 (1.6)	3/356 (0.8)	1.87 (0.30–11.40)	0.73
Staged or urgent repeat revascularization, *n*/*N* (%)	39/248 (15.7)	34/663 (5.1)	3.07 (1.94–4.86)	<0.0001	33/192 (17.2)	20/356 (5.6)	3.06 (1.75–5.40)	<0.0001
Stroke, *n*/*N* (%)	3/248 (1.2)	5/663 (0.8)	1.60 (0.31–7.61)	0.51	3/192 (1.6)	3/356 (0.8)	1.87 (0.30–11.40)	0.73
Outcomes at 12 months								
Death from any cause, *n*/*N* (%)	404/655 (61.7)	592/896 (66.1)	0.93 (0.86–1.01)	0.075	320/512 (62.5)	334/491 (68.0)	0.92 (0.84–1.01)	0.066
Recurrent myocardial infarction, *n*/*N* (%)	19/229 (8.3)	36/641 (5.6)	1.48 (0.83–2.60)	0.15	15/182 (8.2)	18/338 (5.3)	1.55 (0.76–3.15)	0.19
Rehospitalization for congestive heart failure, *n*/*N* (%)	14/229 (6.1)	33/641 (5.1)	1.20 (0.63–2.29)	0.58	12/182 (6.6)	21/338 (6.2)	1.06 (0.50–2.20)	0.87
Stroke, *n*/*N* (%)	5/229 (2.2)	14/641 (2.2)	1.00 (0.32–2.93)	>0.99	4/182 (2.2)	8/338 (2.4)	0.93 (0.24–3.34)	0.99
Repeat revascularization								
Any, *n*/*N* (%)	73/229 (31.9)	91/641 (14.2)	2.25 (1.69–2.96)	<0.0001	60/182 (33.0)	49/338 (14.5)	2.27 (1.61–3.22)	<0.0001
PCI, *n*/*N* (%)	58/229 (25.3)	80/641 (12.5)	2.03 (1.48–2.77)	<0.0001	49/182 (26.9)	43/338 (12.7)	2.12 (1.44–3.12)	<0.0001
CABG, *n*/*N* (%)	16/229 (7.0)	13/641 (2.0)	3.45 (1.60–7.49)	<0.0001	12/182 (6.6)	7/338 (2.1)	3.18 (1.19–8.83)	0.0040

Abbreviations: CABG = coronary artery bypass grafting; PCI = percutaneous coronary intervention.

## Data Availability

Not applicable.
